# Transient Hypocalcemia in a Dialysis Patient With Paget’s disease and Presumed Renal Cell Carcinoma

**DOI:** 10.1177/2324709616640818

**Published:** 2016-03-24

**Authors:** Kenneth R. Phelps, Jay Mo, Chrystina Czerwinskyj, Roy O. Mathew

**Affiliations:** 1 Stratton Veterans Affairs Medical Center, Albany, NY, USA; 2 Albany Medical College, Albany, NY, USA

**Keywords:** hypocalcemia, chronic kidney disease, hemodialysis, kidney cancer

## Abstract

A 68-year-old man with end-stage renal disease was hospitalized because of radicular pain and weakness in the left arm and hand. Sonography and computed tomography had recently shown a large right renal mass. On admission, magnetic resonance imaging demonstrated vertebral metastases with epidural extension, and radiotherapy was directed to the spine and kidney. Hypocalcemia was first noted on the fourth hospital day. A second computed tomography scan showed bleeding into and around the kidney, and arterial embolization was required to halt the bleeding. Hypocalcemia persisted for at least 27 days at values between 6.0 and 7.7 mg/dL and was consistently associated with ionized calcium concentrations less than or equal to 4.44 mg/dL. After an unrevealing search for a recognized cause, we attributed hypocalcemia to persistent sequestration of calcium in the right retroperitoneum. Exogenous supplementation eventually restored the concentration to normal. In the absence of renal and intestinal loss, hypocalcemia reflects abnormal flux of calcium from the extracellular compartment into tissue. Our patient’s repository appears to have been a necrotic and hemorrhagic cancer. Tumor-induced sequestration of calcium should be included in the differential diagnosis of hypocalcemia.

## Introduction

In hemodialysis patients, hypocalcemia has been reported as a complication of extreme hyperphosphatemia, low dialysate calcium, abrupt reduction in the parathyroid hormone concentration ([PTH]), antiresporptive therapy with denosumab, and iron chelation with deferasirox.^1-6^ This report describes a patient with transient hypocalcemia that could not be attributed to recognized causes. We ultimately concluded that a necrotic renal cancer had provided a temporary repository for extracellular calcium.

## Case Report

A 68-year-old man was hospitalized because of radicular left arm pain of 2 weeks duration. Over the previous 14 weeks, the hemoglobin concentration ([hgb]) had fallen from 9.6 g/dL to values as low as 6.6 g/dL; 2 units of red blood cells (RBCs) had been transfused 4 weeks prior to admission. The patient, who was anuric, had experienced a single episode of gross hematuria almost 2 months before admission, but investigation of this complaint had been delayed. Nine days before admission, a renal sonogram had shown an atrophic left kidney and a large complex mass replacing the right kidney.

The patient had hypertensive renal disease and had undergone thrice-weekly hemodialysis for 2¾ years. Paget’s disease had been diagnosed approximately 17 years prior to admission; 8 years before admission a bone scan had shown increased activity in the pelvis, thoracic spine, and right humerus. The serum alkaline phosphatase concentration ([AP]_s_) had subsequently varied between 400 and 1000 units/L. [PTH] had been consistently elevated during the patient’s tenure on dialysis. Excellent control of phosphatemia ([P]_s_) had been achieved with 2 intestinal phosphate binders.

Medicines on admission included calcium acetate 1334 mg and lanthanum carbonate 1000 mg 3 times daily with meals; a multivitamin once daily; folic acid 1 mg daily; metoprolol tartrate 50 mg 3 times daily on nondialysis days; a nutritional supplement 3 times daily; simvastatin 40 mg at bedtime; sodium bicarbonate 650 mg 3 times daily; and ergocalciferol 50 000 units weekly. Cinacalcet had never been prescribed. During each dialysis treatment the patient received heparin, 3000 units at the outset and 1000 units/hour; paricalcitol 5 µg; and sodium ferric gluconate 62.5 mg. He also received darbepoietin 200 µg weekly.

Vital signs on admission were temperature 98.7°F, pulse 86/minute, blood pressure 187/89 mm Hg, and respiratory rate 16/minute. Skin, lymph nodes, lungs, and heart were normal. No masses or organomegaly were detected in the abdomen. Grip strength, wrist dorsiflexion, and elbow extension were weak in the left upper extremity, and pinprick sensation was reduced in the left fifth finger. Leg strength was normal; lower extremity reflexes were not hyperactive, and Babinski reflexes and clonus were absent. The patient was not incontinent.

**Figure 1.**
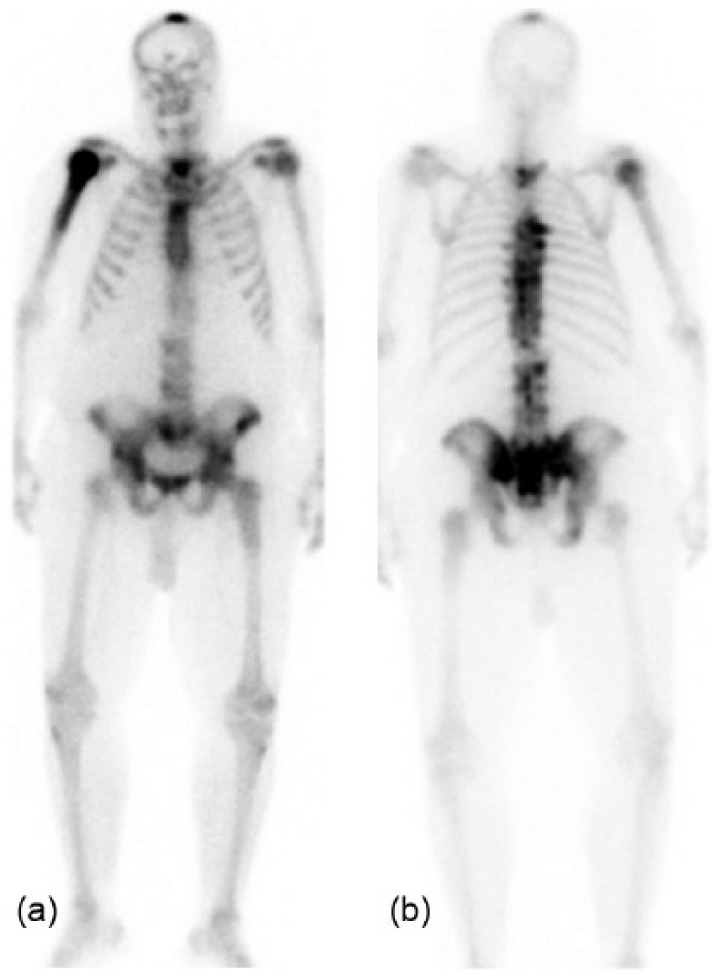
Technetium^99^ medronate bone scan. Images “a” and “b” are anterior and posterior views, respectively. They were obtained 6 days prior to admission, and findings were compared to those described in a report from 2004. Increased uptake in the vertex of the skull was seen in the present study only. Lesions in the right humerus, thoracic vertebrae, and sacrum were described previously.

Results of a technetium medronate bone scan performed 6 days before admission were compared to findings reported 8 years earlier. Previously described areas of increased activity were present in the right humerus, the sacrum, and the thoracic spine. An area of high uptake in the vertex of the skull had not been evident in the earlier study (Figure 1).

**Figure 2.**
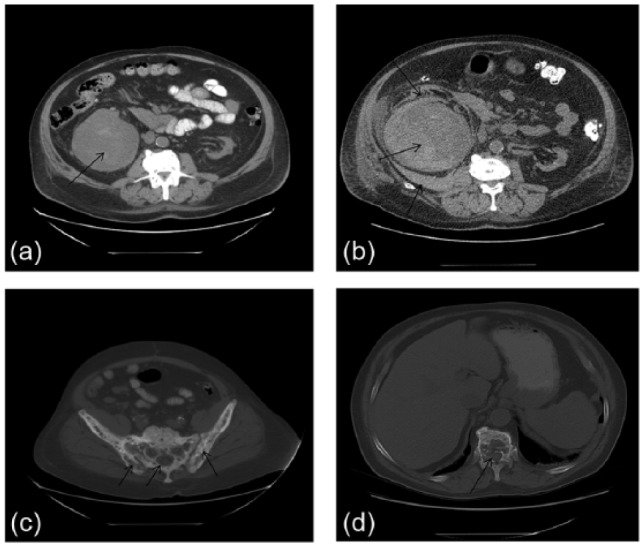
Computed tomography of the abdomen. Images “a,” “c,” and “d” are taken from a study performed on the day prior to admission. Image “b,” which approximates the tomogram in image “a,” is taken from a study done on the fourth hospital day. Image “a” shows a large, complex mass in the right kidney (arrow). Image “c” demonstrates skeletal changes of Paget’s disease in the ilia and sacrum (arrows). Image “d” shows bone lysis in the T12 vertebral body and violation of the cortex adjacent to the spinal canal (arrow). In image “b,” blood has accumulated anterior to, within, and posterior to the mass (arrows).

On the day prior to admission, computed tomography (CT) of the abdomen confirmed the presence of a large, mixed-density mass in the right kidney (Figure 2a). Cortical thickening and increased trabeculation in multiple ribs, thoracic and lumbar vertebrae, the pelvis, and the left femur were suggestive of Paget’s disease (Figure 2c). Bone lysis and cortical breakthrough in T11 and T12 were attributed to malignancy (Figure 2d). Adenopathy was not seen.

**Figure 3.**
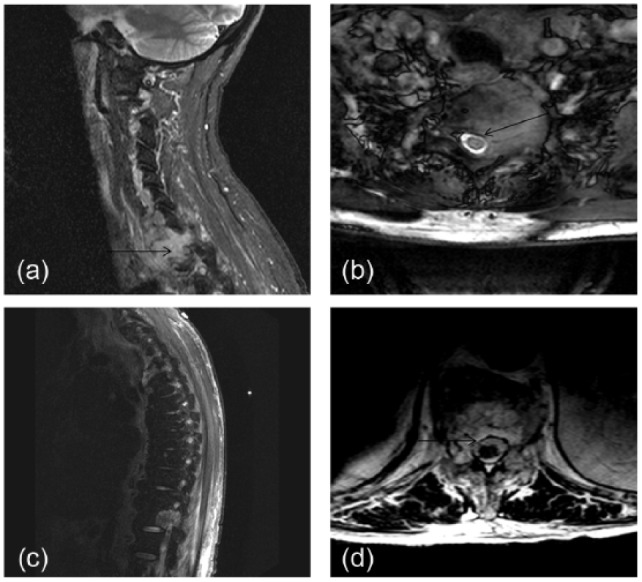
Magnetic resonance imaging of the cervical and thoracic spine. Images are taken from a study performed on the day of admission. Image “a” is a left parasagittal view of the cervical spine showing an expansile mass (arrow) in the bodies of C7 and T1 with a high T2 signal. Image “b” is a cross-sectional view of T1 showing encroachment on the vertebral canal by the lesion (arrow). Image “c” shows a similar lesion at T12. Image “d” demonstrates encroachment of this mass on the vertebral canal (arrow).

On the day of admission, magnetic resonance imaging of the spine showed an expansile mass involving C7 and T1 with cortical breakthrough, epidural extension, and effacement of the left C7-T1 neural foramen. Cord compression was not evident. At T12, another expansile lesion involved the posterior vertebral body and the right pedicle and extended into the right anterior spinal canal. Widespread marrow signal changes and numerous focal lesions were suggestive of extensive vertebral metastases (Figure 3).

After administration of 10 mg intravenously, dexamethasone was prescribed at an oral dose of 4 mg every 6 hours in anticipation of radiotherapy, which was begun on the second hospital day. In total, 32 Gy was delivered over 10 treatments to the metastasis at C7-T1, and 30 Gy to the metastasis at T12 and the renal mass.

On admission, [hgb] was 6.2 g/dL. Three units of packed RBCs were administered on the third and fourth hospital days, but [hgb] fell to a nadir of 5.6 g/dL. A second computed tomography scan confirmed the presence of blood within, anterior to, and posterior to the mass (Figure 2b), and the patient was referred for right renal arterial embolization. Three additional units of RBCs were infused over the ensuing 2 days, and administration of heparin during dialysis was discontinued. Transfusions were then withheld for 25 consecutive days.

**Figure 4.**
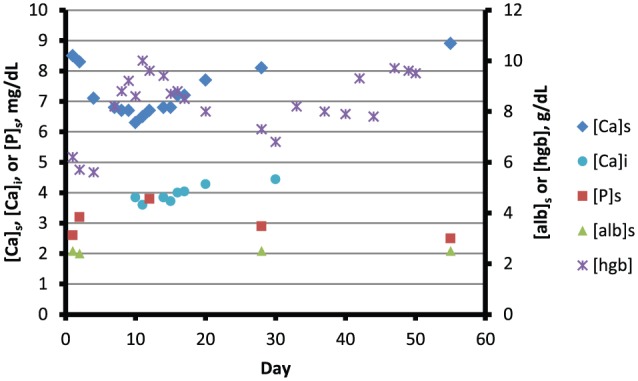
Blood hemoglobin and parameters of mineral metabolism over a 60-day period. The period commences with the hospital admission described in the text. Radiotherapy was initiated on day 2, and arterial embolization of the right kidney was performed on day 4. The nadir of [Ca]_s_ was reached gradually over 10 days, and was associated with persistent reductions in [Ca]_i_. [Ca]_i_ was partially restored at day 30. [Ca]_s_ was found to be normal at day 60. Abbreviations: [Ca]_s_, total serum calcium concentration; [Ca]_i_, serum ionized calcium concentration; [P]_s_, serum phosphorus concentration; [alb]_s_, serum albumin concentration; [hgb], blood hemoglobin concentration. Reference ranges: [Ca]_s_, 8.5-10.2 mg/dL; [Ca]_i_, 4.72-5.28 mg/dL; [P]_s_, 2.5-4.5 mg/dL; [alb]_s_, 3.4-4.5 g/dL; [hgb], 13.5-17 g/dL.

Table 1 shows temporal relationships between laboratory data and therapeutic efforts during a 30-day period commencing on day 1. Figure 4 depicts serum concentrations of total calcium ([Ca]_s_), ionized calcium ([Ca]_i_), phosphorus ([P]_s_), and albumin ([alb]_s_), and the blood concentration of hemoglobin ([hgb]) over a 60-day period commencing on the same day. Reference ranges are provided in the legend. On day 2, the first day of radiotherapy, [Ca]_s_, [alb]_s_, and [P]_s_, were 8.3 mg/dL, 2.4 g/dL, and 3.2 mg/dL, respectively. On day 4, [Ca]_s_ was 7.1 mg/dL, and over the next 5 days it fell to 6.3 mg/dL, with a commensurate reduction in [Ca]_i_. Manifestations of hypocalcemia did not appear.

**Table 1.** Overview of Hospitalization^a^.

Day^b^	[Ca], mg/dL (8.5-10.2)	[alb], g/dL (3.4-4.5)	[Ca]_i_, mg/dL (4.72-5.28)	[P], mg/dL (2.5-4.5)	[PTH], pg/mL (18.6-87.8)	[25D], ng/mL (30-100)	AP, U/L (50-136)	[hgb], g/dL (13.5-17)	Units RBC	HD	PO Ca, mg/day^c,d^	PO 1,25D, µg/day^d^	IV Ca, mg/day^e^	RT
1	8.5	2.5		2.6			195	6.2		x				
2	8.3	2.4		3.2				5.7						x
3	7.1					46.3			2	x				x
4^f^								6.1/5.6	1					
5									3					
6										x				
7	6.8							8.2						x
8	6.7							8.8		x				x
9	6.7							9.2						x
10	6.3		3.84					8.6		x				x
11	6.5		3.60		673.2			10.0					279	
12	6.7			3.8				9.6			780	0.25	186	
13										x	780	0.25		
14	6.8		3.84					9.4			780	0.5		x
15	6.8		3.72					8.7		x^g^	780	0.5		x
16	7.2		4.00					8.8			780	0.5		
17^b^	7.2		4.04				146	8.5		x^g^	780	0.5		x
20	7.7		4.28					8.0		x^g^	780	0.5		x
28	8.1	2.5		2.9	467.5	51.5		7.3		x	1500	0.5		
30			4.44					6.8	2	x	1500^h^	0.5^i^		

Abbreviations: Ca, calcium; alb, albumin; Ca_i_, ionized calcium; P, phosphorus; PTH, parathyroid hormone; 25D, 25-hydroxyvitamin D; 1,25D, 1,25-dihydroxyvitamin D3 (calcitriol); AP, alkaline phosphatase; hgb, hemoglobin; RBC, red blood cells; HD, hemodialysis treatment; RT, radiotherapy treatment. Doses and sites of RT are provided in the text.

a Concentrations are in serum. Normal ranges are given in parentheses.

b Enumerated from day of admission to hospital. Discharge was on day 17.

c Given as calcium carbonate.

d Daily administration continued after discharge.

e Given as calcium gluconate.

f Date of right renal arterial embolization.

g Dialysis these days against [Ca] 6.0 mg/dL, and against 5.0 mg/dL on all other days. Dialysis continued on a thrice-weekly schedule after discharge.

h Dose still in effect on day 60.

i Dose expired on day 46.

Calcium and ergocalciferol were not administered during the first 10 days of hospitalization, and cinacalcet was not provided at any time. Paricalcitol was not given during the periods summarized in Table 1 and Figure 4. Hemodialysis treatments were performed thrice weekly for 4 hours with Asahi Rexeed-18 high-flux polysulfone dialyzers. The dialysate calcium concentration was 5.0 mg/dL on all but 3 occasions; on days 15, 17, and 20 it was 6.0 mg/dL.

**Figure 5.**
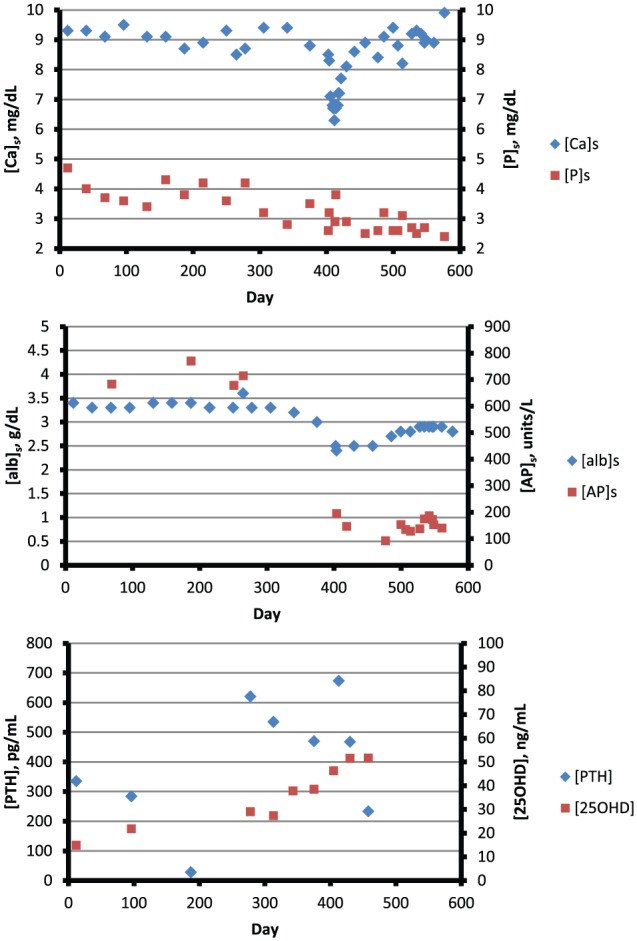
Parameters of mineral metabolism over a 600-day period. The period commences approximately 400 days prior to admission. During those 400 days, [Ca]_s_ and [P]_s_ were normal. [AP]_s_ consistently exceeded 600 units/L, but approximated the upper limit of normal during and after hospitalization. [25OHD]_s_ was brought into the normal range before admission. [PTH] was consistently increased to several times the upper limit of normal. Abbreviations: [AP]_s_, serum alkaline phosphate concentration; [25OHD], serum 25-hydroxyvitamin D concentration; [PTH], serum PTH concentration. Other abbreviations are provided in the legend for Figure 4. Reference ranges are provided in Figure 4 and Table 1.

On day 11, 279 mg of calcium was infused as calcium gluconate; on day 12, an additional 186 mg was infused, and oral calcium carbonate was added with meals in a dose of 650 mg 3 times daily. Calcitriol, 0.25 µg/day, was prescribed on day 11 and increased to 0.25 µg twice daily on day 14. Introduction of these supplements was followed by a gradual rise in [Ca]_i_; the concentration was 4.04 mg/dL on day 17 (the date of discharge), 4.28 mg/d L on day 20, and 4.44 mg/dL on day 30. Thereafter, for a period of several months, [Ca]_s_ exceeded 8.0 mg/dL while [alb]_s_ remained at or slightly below 3.0 g/dL (Figure 5).

Tests to investigate the cause of hypocalcemia were performed between the 3rd and 17th hospital days (Table 2). Serum concentrations of magnesium ([Mg]_s_), phosphorus ([P]_s_), urate ([ur]_s_), and 25-hydroxyvitamin D ([25OHD]) were normal, and concentrations of parathyroid hormone ([PTH]) and N-telopeptide ([NTx]) were markedly elevated. [AP]_s_ was minimally increased. Reference ranges and conditions sought are summarized in Table 2.

**Table 2.** Laboratory Investigations to Determine the Cause of Hypocalcemia^a^.

Test	Condition Sought	Hospital Day	Result	Reference Range
[P], mg/dL	Tumor lysis syndrome	12	3.8	2.5-4.5
[ur], mg/dL	Tumor lysis syndrome	12	4.1	3.9-9.0
[Mg], mg/dL	Hypomagnesemia	15	2.0	1.9-2.7
[25OHD], ng/mL	Vitamin D deficiency	3	46.3	30.0-100.0
[PTH], pg/mL	Hypoparathyroidism	11	673.2	18.6-87.8
[AP], units/L	Excessive bone formation	17	146	50-136
[NTx], BCE/L	Suppressed bone resorption	17	417.7	5.4-24.2

Abbreviations: P, phosphorus; ur, urate; Mg, magnesium; 25OHD, 25-hydroxyvitamin D; PTH, parathyroid hormone; AP, alkaline phosphatase; NTx, N-telopeptide; BCE, bone collagen equivalents.

a All determinations in serum.

Figure 5 provides a 600-day temporal perspective. Over approximately 400 days prior to admission, [PTH] was consistently elevated despite normal [P]_s_ and administration of parenteral paricalcitol during dialysis treatments. [25OHD] was robustly normal in response to supplemental ergocalciferol. [Alb]_s_ remained between 3.0 and 3.5 g/dL, but it fell to a nadir of 2.4 g/dL during the period of hypocalcemia. [AP]_s_ was consistently increased until 5 months before admission, but all subsequent values approximated or slightly exceeded the upper limit of normal.

The clinical suspicion of metastatic cancer was not confirmed histologically. An earlier iliac crest biopsy had shown no malignant cells, and risks associated with a renal or vertebral biopsy seemed prohibitive. Sunitinib was administered after completion of radiotherapy, but pulmonary and other metastases continued to appear and expand. The patient died in December 2012. An autopsy was not performed.

## Discussion

This report describes a transient episode of hypocalcemia in a hemodialysis patient presumed to have renal cell cancer and metastases to bone. Measurements of [Ca]_i_ repeatedly confirmed the hypocalcemia. The abnormality appeared after initiation of radiotherapy to the spine and right kidney and persisted for at least 27 days. It responded minimally to intravenous calcium during days 11 and 12, and gradually but incompletely to dialysis and oral calcium and calcitriol during days 12 to 30. The time required to restore normocalcemia was less than 60 days but is not precisely known.

During the first 10 days of hospitalization, we presume that omission of calcium acetate and paricalcitol from the medical regimen reduced intestinal calcium absorption. Simultaneously, however, we suspect that acquisition of calcium during dialysis equaled or exceeded this reduction. Values of [Ca]_i_ on days 10 and 11 imply that dialysate-to-plasma [Ca] gradients had exceeded 1.0 mg/dL during the preceding week. On average, during a 4-hour dialysis treatment, such gradients should have promoted diffusion of at least 400 mg of calcium into plasma as 140 mg was removed by convection, with a net gain to the patient of 260 mg per treatment.^7^ Because the patient had no diarrhea and produced no urine, it is unlikely that calcium was lost externally at a comparable rate if it was lost at all. During the period when [Ca]_s_ was stable at its nadir, we infer that calcium accumulated in tissue at the rate of net influx.

On days 11 and 12, intravenous infusion of 465 mg of calcium had a negligible effect on [Ca]_i_. Between days 12 and 30, dialysis against physiologic or higher [Ca] and supplementation with calcium and calcitriol raised [Ca]_i_ gradually but failed to correct it (Table 1). During days 1 to 30, we estimate that at least 2000 mg of exogenous calcium accumulated in a tissue reservoir.

We sought other causes of hypocalcemia. The patient presented no clinical evidence of pancreatitis or myonecrosis.^8,9^ Chelation by citrate could have reduced [Ca]_i_ during RBC transfusions;^10^ whereas [Ca]_s_ typically remains normal during this process,^11^ [Ca]_s_ and [Ca]_i_ fell in tandem in our patient (Figure 4), and hypocalcemia persisted for many days in the absence of transfusions.

We considered the possibility that dexamethasone had caused hypocalcemia. Steroids have lowered [Ca]_s_ by causing pancreatitis or tumor lysis,^12,13^ or by interfering with intestinal calcium absorption in patients with hypoparathyroidism or vitamin D deficiency.^14,15^ However, under ordinary circumstances, exogenous steroids inhibit bone formation while resorption continues unabated, and the skeleton incurs a net loss of calcium.^16^ We conclude that dexamethasone did not reduce [Ca]_s_ in our patient.

Although chemotherapy had not been administered when hypocalcemia appeared, we wondered whether tumor lysis due to radiotherapy had lowered [Ca]_s_. We measured [P]_s_ and [ur]_s_ to investigate this possibility,^17^ but neither was elevated. Hypomagnesemia may limit secretion of and end-organ responsiveness to PTH,^18,19^ but [Mg]_s_ was also normal.

We considered the possibility that the skeleton had become an acutely receptive repository for calcium. In both Paget’s disease and secondary hyperparathyroidism (SHPT), rapid turnover of bone may produce an excess of unmineralized osteoid.^20,21^ We therefore contemplated a scenario, similar to the post-parathyroidectomy hungry-bone syndrome,^22^ in which calcium flux from the skeleton had fallen while mineralization of osteoid continued unabated. We speculated that radiation-induced damage to bone or to the parathyroid glands had created the hypothetical disequilibrium.

Test results did not support this speculation. Although the concentration of alkaline phosphatase, a marker of bone formation, had been 714 units/L 5 months prior to admission, it was only 195 units/L on hospital day 2, and it fell further during the ensuing month (Table 1; Figure 2). In contrast, the concentration of N-telopeptide, a marker of bone resporption, was found to be markedly increased (Table 2), and [PTH] remained elevated despite radiotherapy to C7 and T1. The limited fields of radiation and the implied discrepancy between resorption and formation suggest that the skeleton was an unlikely repository for calcium. Although pharmacotherapy of SHPT or Paget’s disease may lower [Ca]_s_,^3,23^ we can find no evidence that radiation of affected bone has produced this result. We presume that lytic metastases caused net calcium flux into plasma.

Clinical and laboratory findings suggest that hypocalcemia did not result from drug toxicity, myonecrosis, pancreatitis, hypoparathyroidism, vitamin D deficiency, hypomagnesemia, tumor lysis syndrome, or hungry-bone syndrome. Because dialysate calcium levels were physiologic or higher, the only potential avenues of calcium loss from extracellular fluid were intestinal secretion and tissue deposition. We do not believe that muscle, pancreas, or bone served as a repository.

Our patient’s [Ca]_s_ had remained consistently normal before admission. Hypocalcemia was detected 2 days after the initiation of radiotherapy, which appeared to exacerbate necrosis of and hemorrhage into a renal cancer. Arterial embolization probably amplified the necrosis. [Ca]_i_ rose minimally with an intravenous infusion of calcium during days 11 and 12; through day 30, [Ca]_i_ responded gradually but incompletely to influx of hundreds of milligrams of calcium from oral supplements and dialysate. We propose that a large collection of tissue and blood provided a reservoir for extracellular calcium; [Ca]_s_ fell to a level at which influx from all sources could compensate for efflux into the right retroperitoneum, and returned to normal when the reservoir could accept no more calcium. In extraordinary circumstances, it appears that extensive tumor necrosis and associated hemorrhage can cause hypocalcemia.

## References

[1] Mishra R Kaufman D Mattern J 3rd Dutta SK. Severe hyperphosphatemia and hypocalcemia caused by bowel preparation for colonoscopy using oral sodium phosphate in end-stage renal disease. Endoscopy. 2005;37:1259-1260. 16329033 10.1055/s-2005-921155

[2] Ulozas E Chebrolu SB Shanaah A Daoud TM Leehey DJ Ing TS. Symptomatic hypocalcemia due to the inadvertent use of a calcium-free hemodialysate. Artif Organs. 2004;28:229-231. 14961964 10.1111/j.1525-1594.2004.47207.x

[3] Ketteler M Martin KJ Wolf M Paricalcitol versus cinacalcet plus low-dose vitamin D therapy for the treatment of secondary hyperparathyroidism in patients receiving haemodialysis: results of the IMPACT SHPT study. Nephrol Dial Transplant. 2012;27:3270-3278. 22387567 10.1093/ndt/gfs018 PMC3408938

[4] Stracke S Jehle PM Sturm D Clinical course after total parathyroidectomy without autotransplantation in patients with end-stage renal failure. Am J Kidney Dis. 1999;33:304-311. 10023643 10.1016/s0272-6386(99)70305-7

[5] McCormick BB Davis J Burns KD. Severe hypocalcemia following denosumab injection in a hemodialysis patient. Am J Kidney Dis. 2012;10:626-628. 22854051 10.1053/j.ajkd.2012.06.019

[6] Yusuf B McPhedran P Brewster UC. Hypocalcemia in a dialysis patient treated with deferasirox for iron overload. Am J Kidney Dis. 2008;52:587-590. 18534729 10.1053/j.ajkd.2008.03.034

[7] Bosticardo G Malberti F Basile C Optimizing the dialysate calcium concentration in bicarbonate hemodialysis. Nephrol Dial Transplant. 2012;27:2489-2496. 22357700 10.1093/ndt/gfr733

[8] Weir GC Lesser PB Drop LJ Fischer JE Warshaw AL. The hypocalcemia of acute pancreatitis. Ann Intern Med. 1975;83:185-189. 1147452 10.7326/0003-4819-83-2-185

[9] Llach F Felsenfeld AJ Haussler MR. The pathophysiology of altered calcium metabolism in rhabdomyolysis-induced acute renal failure. N Engl J Med. 1981;305:117-123. 6894630 10.1056/NEJM198107163050301

[10] Sihler KC Napolitano LM. Complications of massive transfusion. Chest. 2010;137:209-220. 20051407 10.1378/chest.09-0252

[11] Kahn RC Jascott D Carlon GC Schweizer O Howland WS Goldiner PL. Massive blood replacement: correlation of ionized calcium, citrate, and hydrogen ion concentration. Anesth Analg. 1979;58:274-278. 36816 10.1213/00000539-197907000-00003

[12] Cortese AF Glenn F. Hypocalcemia and tetany with steroid-induced acute pancreatitis. Arch Surg. 1968;96:119-122. 4294942 10.1001/archsurg.1968.01330190121027

[13] Malik IA Abubakar S Alam F Khan A. Dexamethasone-induced tumor lysis syndrome in high-grade non-Hodgkin’s lymphoma. South Med J. 1994;87:409-411. 8134869 10.1097/00007611-199403000-00024

[14] Vardi P Benderly AS Etzioni A Levy J Hochberg Z. Hypocalcaemia induced by glucocorticoids in a child with hypoparathyroidism treated with 1-alpha-hydroxyvitamin D3. Eur J Pediatr. 1985;144:280-282. 4054169 10.1007/BF00451962

[15] Kinoshita Y Masuyoka K Miyakoshi S Taniguchi S Takeuchi Y. Vitamin D insufficiency underlies unexpected hypocalcemia following high dose glucocorticoid therapy. Bone. 2008;42:226-228. 17964238 10.1016/j.bone.2007.09.042

[16] Dovio A Perazzolo L Osella G Immediate fall of bone formation and transient increase of bone resorption in the course of high-dose, short-term glucocorticoid therapy in young patients with multiple sclerosis. J Clin Endocrinol Metab. 2004;89:4923-4928. 15472186 10.1210/jc.2004-0164

[17] Wilson FP Berns JS. Tumor lysis syndrome: new challenges and recent advances. Adv Chronic Kidney Dis. 2014;21:18-26. 24359983 10.1053/j.ackd.2013.07.001 PMC4017246

[18] Mennes P Rosenbaum R Martin K Slatopolsky E. Hypomagnesemia and impaired parathyroid hormone secretion in chronic renal disease. Ann Intern Med. 1978;88:206-209. 626450 10.7326/0003-4819-88-2-206

[19] Chase LR Slatopolsky E. Secretion and metabolic efficacy of parathyroid hormone in patients with severe hypomagnesemia. J Clin Endocrinol Metab. 1974;38:363-371. 4360918 10.1210/jcem-38-3-363

[20] Seitz S Priemel M Zustin J Paget’s disease of bone: histologic analysis of 754 patients. J Bone Miner Res. 2009;24:62-69. 18767930 10.1359/jbmr.080907

[21] Urena Torres P Bover J Mazzaferro S de Vernejoul MC Cohen-Solal M. When, how, and why a bone biopsy should be performed in patients with chronic kidney disease. Semin Nephrol. 2014;34:612-625. 25498380 10.1016/j.semnephrol.2014.09.004

[22] Florescu MC Islam KMM Plumb TJ Smith-Shull S Nieman J Mandalapu P. Calcium supplementation after parathyroidectomy in dialysis and renal transplant patients. Int J Nephrol Renovasc Dis. 2014;7:183-190. 24868170 10.2147/IJNRD.S56995 PMC4027938

[23] Polyzos SA Anastasilakis AD Makras P Terpos E. Paget’s disease of bone and calcium homeostasis: focus on bisphosphonate treatment. Exp Clin Endocrinol Diabetes. 2011;119:519-524. 21811962 10.1055/s-0031-1284365

